# Water Extract of Ashwagandha Leaves Limits Proliferation and Migration, and Induces Differentiation in Glioma Cells

**DOI:** 10.1093/ecam/nep188

**Published:** 2011-02-14

**Authors:** Hardeep Kataria, Navjot Shah, Sunil C. Kaul, Renu Wadhwa, Gurcharan Kaur

**Affiliations:** ^1^Department of Biotechnology, Guru Nanak Dev University, Amritsar 143005, India; ^2^National Institute of Advanced Industrial Science & Technology (AIST), Central 4, 1-1-1 Higashi, Tsukuba, Ibaraki 305-8562, Japan

## Abstract

Root extracts of *Withania somnifera* (Ashwagandha) are commonly used as a remedy for a variety of ailments and a general tonic for overall health and longevity in the Indian traditional medicine system, Ayurveda. We undertook a study to investigate the anti-proliferative and differentiation-inducing activities in the water extract of Ashwagandha leaves (ASH-WEX) by examining in glioma cells. Preliminary detection for phytochemicals was performed by thin-layer chromatography. Cytotoxicity was determined using trypan blue and MTT assays. Expression level of an hsp70 family protein (mortalin), glial cell differentiation marker [glial fibrillary acidic protein (GFAP)] and neural cell adhesion molecule (NCAM) were analyzed by immunocytochemistry and immunoblotting. Anti-migratory assay was also done using wound-scratch assay. Expression levels of mortalin, GFAP and NCAM showed changes, subsequent to the treatment with ASH-WEX. The data support the existence of anti-proliferative, differentiation-inducing and anti-migratory/anti-metastasis activities in ASH-WEX that could be used as potentially safe and complimentary therapy for glioma.

## 1. Introduction

One of the most versatile plants used in the traditional Indian medicine system (Ayurveda) is *Withania somnifera* (Ashwagandha). It is highly reputed as “Indian ginseng" and is a member of generally regarded as safe (GRAS) plants [1]. Extracts from different parts of Ashwagandha have been claimed to promote physical and mental health. The biologically active constituents of Ashwagandha include alkaloids, steroidal lactones and saponins [2] that have been shown to possess neurite outgrowth and anti-cancer activities [3]. Withanone present in alcoholic extract of Ashwagandha leaves was shown to selectively kill cancer cells by activation of tumor suppressor protein p53 [4].

Glioma is highly invasive primary brain tumor that awaits therapeutics. Extracts of Ashwagandha have been reported to have inhibitory effects on different types of cancers in animal models and on cell lines [4, 5]. However, there is very little information on their effect on brain tumors and tumor-derived cell lines. Since C6 glioma, an *N*-nitrosomethyl-urea-induced rat glioma, shows normal glial cell properties, it has been extensively used as an *in vitro* glial model system [6]. We examined the expression of several proteins including heat shock 70 family protein (mortalin), immunoglobulin superfamily neural cell adhesion molecule (NCAM) and an intermediate filament protein (glial fibrillary acidic protein, GFAP) that depicted the proliferative, tumorigenic, adhesion and differentiation characteristics [7–9] in control and water extract of Ashwagandha leaves (ASH-WEX)-treated cells. Although mortalin expression and its subcellular localization showed the proliferative characteristics of cells, GFAP and NCAM were used as markers of the differentiation characteristics including morphogenesis, neural cell differentiation, axonal outgrowth and fasciculation [10]. We present the first evidence of the anti-proliferative activity of ASH-WEX that is closely associated with the induction of senescence and differentiation in glioma cells.

## 2. Methods

### 2.1. Preparation of ASH-WEX

Dry powder of Ashwagandha leaves was obtained from the Department of Botanical and Environmental Sciences, Guru Nanak Dev University, Amritsar, India, courtesy of Dr A. Nagpal. ASH-WEX was prepared by suspending 10 g of dry leaf powder in 100 ml of distilled water and stirring it overnight at 45 ± 5°C, followed by filtration under sterile conditions. The filtrate thus obtained was treated as 100% ASH-WEX. It was stored at –20°C in 1 ml aliquots until further use.

### 2.2. Cell Culture and ASH-WEX Treatments

Rat C6 glioma cell line was obtained from NCCS, Pune, India. Human glioma cell lines—YKG1, U118MG and A172—were obtained from Health Science Research Resources Bank, Japan. The cell lines were maintained on Dulbecco's modified Eagle's minimal essential medium (DMEM)—supplemented with streptomycin (100 U ml^−1^), gentamycin (100 *μ*g ml^−1^), 10% FCS (Himedia) at 37°C and humid environment containing 5% CO_2_. Cultures at 30–40% confluency were treated with ASH-WEX (0.1–2.5% diluted in medium) for 72 hours. The medium of control culture was replaced with a fresh one.

### 2.3. Chemical Standardization of ASH-WEX and Nature of Active Components

ASH-WEX was subjected to preliminary phytochemical screening for alkaloids, amino acids, anthraquinones, flavonoids, phytosterols, saponins, steroids, tannins, triterpenoids and reducing sugars following the methods of Harborne [11] and Kokate [12]. It was further subjected to thin-layer chromatography (TLC) using chloroform:methanol (1 : 1) as solvent front. TLC plate was subjected to UV radiation and iodine vapors for observation.

ASH-WEX was also subjected to heat (95°C), protein (proteinase K, 1 mg ml^−1^ and trypsin 1 mg ml^−1^) and nucleotide (DNase I, 100 *μ*g ml^−1^; RNase, 100 *μ*g ml^−1^) inactivation for 30 minutes each and chilled immediately on ice. The control samples were kept on ice during the treatments. These extracts were then tested for anti-proliferative and differentiation-inducing activities.

### 2.4. Proliferation and Cell Membrane Integrity Assays

ASH-WEX was tested for anti-proliferative activity on C6 glioma cells using the 3-(4,5-dimethylthiazol-2-yl)-2, 5-diphenyltetrazolium bromide (MTT) test. In order to assay membrane integrity and viability, growth curves were obtained using trypan blue dye staining method.

### 2.5. Cellular and Nuclear Morphology Studies

Morphological changes in glioma cells treated with different concentrations of ASH-WEX were examined by phase contrast microscopy, and the nuclear morphology was studied using Hoechst 33258 fluorescent microscopy.

### 2.6. Immunostaining

Cells, control and treated, were fixed with paraformaldehyde followed by permeabilization with 0.3% Triton-X 100 in phosphate buffered saline (PBST). Cells were incubated with anti-GFAP (1 : 500, Sigma), anti-mortalin (1 : 500) [13] and anti-NCAM (1 : 500, Abcys) antibodies diluted in 0.1% PBST, for 24 hours at 4°C in humid chamber. Secondary antibody (goat anti-mouse/anti-rabbit IgG FITC conjugated, from Sigma) was applied for 2 hours at room temperature. Cells were then mounted with anti-fading reagent and observed. Images were captured using Cool Snap CCD camera and the pictures were analyzed using image pro-plus software version 4.5.1 from the media cybernetics.

### 2.7. Protein Assay and Western Blotting

Cells grown and treated in 90-mm petridishes were harvested with PBS–EDTA (1 mM). Cell pellet was homogenized in buffer (50 mM Tris, 150 mM NaCl, 1 mM EDTA, 100 *μ*M NaVO_4_, 1 mM PMSF and 0.5 mM DTT) and protein content in the supernatant was determined by the Bradford method.

Protein lysate (20–30 *μ*g) was resolved on 10% sodium dodecyl sulfate-polyacrlylamide gel electrophoresis (SDS-PAGE) [14] followed by blot transfer onto a PVDF membrane (Hybond-P) using the semidry Novablot system (Amersham Pharmacia). Subsequently, membranes were probed with mouse anti-GFAP (1 : 3000), anti-mortalin (1 : 1000) or anti-NCAM (1 : 1000) monoclonal antibodies for 2 hours. Immunoreactive bands were visualized using ECL Plus western blot detection system (Amersham Biosciences). In order to account for potential variations in protein estimation and sample loading, expression of each protein was compared with that of *α*-tubulin in each sample after stripping the blot.

### 2.8. Wound Scratch Assay

In order to investigate C6 cell migration capability, cells were grown to confluent monolayer. Monolayer was wounded by scratching the surface with a needle. Following the treatment, the initial wounding and the movement of cells in the scratched area were photographically monitored for 6 hours after the treatment. To evaluate the correlation between NCAM protein expression and cell migration capability, cells were immunostained with anti-NCAM antibody.

### 2.9. Statistical Analysis

Values are expressed as mean ± SEM. The SigmaStat for Windows (version 3.5) was adopted to analyze the results by Student's *t*-test in order to determine the significance of the means. Values of *P* < .05 were considered as statistically significant.

## 3. Results

### 3.1. Effect of Ashwagandha ASH-WEX on the Proliferation of Glioma Cells

**Figure 1:**
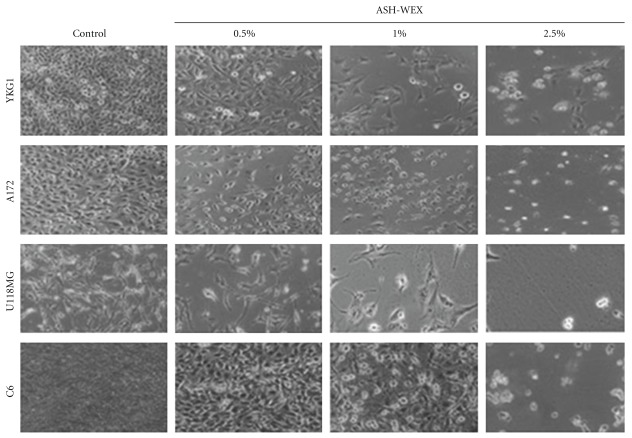
Phase contrast images of C6, U118MG, A172 and YKG1 glioma cells treated with 0.0% (control), 0.5, 1.0 and 2.5% ASH-WEX. There was significant difference in cell number and morphology in treated groups as compared with the control. ASH-WEX-treated cultures showed multiple processes and more of differentiated morphology. Treatment group (2.5%) showed cytotoxic effect of ASH-WEX with majority dead cells.

**Figure 2:**
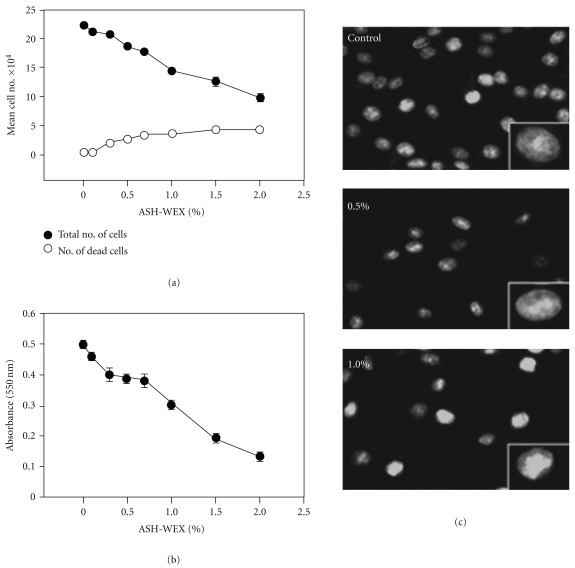
Growth curve inhibition and cytotoxicity assessed by (a) Trypan blue dye uptake assay, (b) MTT assay and (c) Hoechst staining. Data are representative of three different experiments done in triplicates and expressed as mean ± SEM.

Human (YKG1, A172, U118MG) and rat (C6) glioma cell lines were cultured in the presence of different concentrations of ASH-WEX. At higher concentrations (>1%) it caused cytotoxicity and cell death (Figure 1). Cells rounded up in about 48 hours and seemed to undergo apoptotic cell death. However, at lower concentrations (≤0.5%), cells appeared to be growth arrested and showed morphology that appeared similar to the differentiated cells. All the cell lines tested showed similar level of killing at higher concentrations and differentiation-like phenotype at lower concentrations. For ease of culture, we focused the further study on C6 glioma cells. Relationships between the concentrations of ASH-WEX and their anti-proliferative effects on C6 glioma cells were further investigated by MTT assay and manually counting the live and dead cells with hemocytometer. Cells were treated with ASH-WEX at concentrations ranging from 0 to 2.5% for 72 hours and the cell viability was analyzed. ASH-WEX inhibited the proliferation of C6 glioma cells in a dose-dependent manner (Figure 2(a)). The extract limited the proliferation of C6 glioma cells by 5–75% with an IC50 (concentrations of extracts leading to 50% inhibition of cell growth) of about 1.25% (Figure 2(b)). Cytotoxic effects of the extract on C6 glioma cell line were confirmed by trypan blue dye exclusion assay.

### 3.2. ASH-WEX Induces Apoptosis at High Concentration

When the cells were treated with 0.5 and 1% ASH-WEX we found some cells showing bright, fragmented and condensed nuclei, membrane blebbing and apoptotic bodies as seen by Hoechst staining (Figure 2(c)). The data suggested that these concentrations may induce apoptosis in small fraction of cell population when the majority of the cells showed differentiation-type morphology (Figure 1). Of note, the control cells were also seen to have some apoptotic cells. Based on these data, we selected 0.5–1.0% ASH-WEX for its differentiation potential for C6 cells, whereas the concentration >2% caused apoptosis.

### 3.3. Possible Nature of Bioactive Components of ASH-WEX

**Figure 3:**
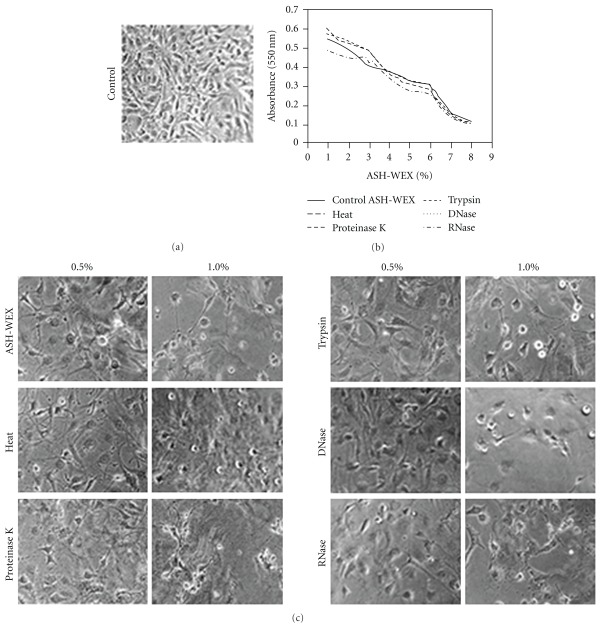
Effect of heat and enzymatic treatments on the anti-proliferative activity of ASH-WEX. The ASH-WEX was treated with heat (95°C), proteinase K (1 mg ml^−1^), trypsin (1 mg ml^−1^), DNase I (100 *μ*g ml^−1^) and RNase (100 *μ*g ml^−1^) for 30 minutes and its anti-proliferative and differentiation-inducing activity at concentrations ranging from 0.1 to –2.0% was evaluated in C6 glioma cells. Phase contrast images of cells (control (a)), and in the presence of heat and enzymatically treated ASH-WEX (c) showed no significant difference in the cell morphology. MTT assay of control and treated ASH-WEX showed similar anti-proliferative activity (b).

**Figure 4:**
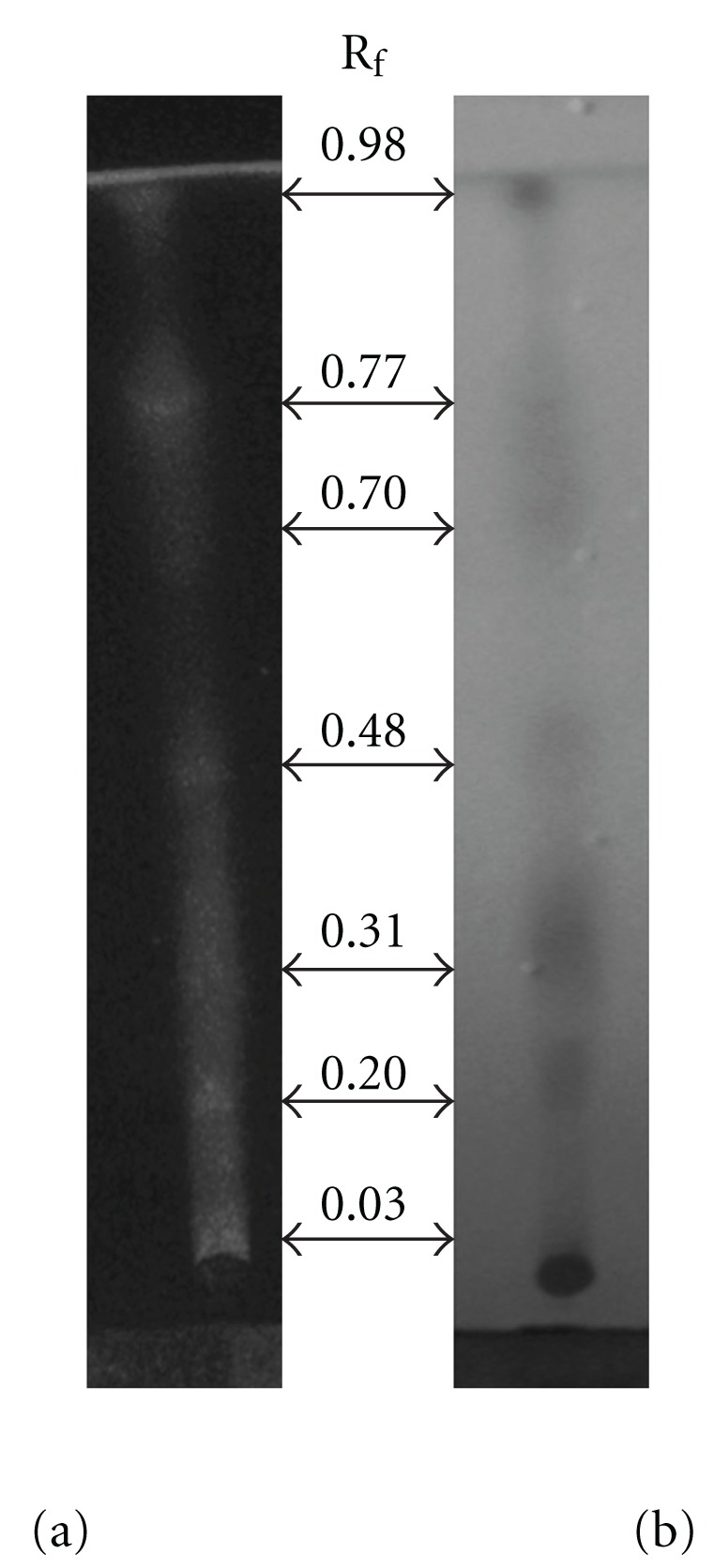
Analysis of ASH-WEX by TLC. Chloroform : methanol (1 : 1) solvent system revealed seven different spots. These spots were observed under (a) UV light and later visualized using (b) iodine vapors.

We inactivated ASH-WEX with heat, proteinase K, trypsin, DNase and RNase and tested its activities. MTT assays and morphological observations (Figure 3) showed no significant difference in anti-proliferative or differentiation-induction effects between control and the cells treated with these inactivated ASH-WEX preparations. Furthermore, preliminary screening tests for phytochemicals showed the presence of flavanoids, steroids, tannins, amino acids, saponins, reducing sugars and alkaloids in ASH-WEX (Table 1). The TLC profile generated by methanol:chloroform (1 : 1) solvent showed the presence of seven spots with Rf values 0.03, 0.20, 0.31, 0.48, 0.70, 0.77 and 0.98 (Figure 4). 

### 3.4. Induction of Differentiation and Senescence-Like Phenotypes in Glioma Cells

**Figure 5:**
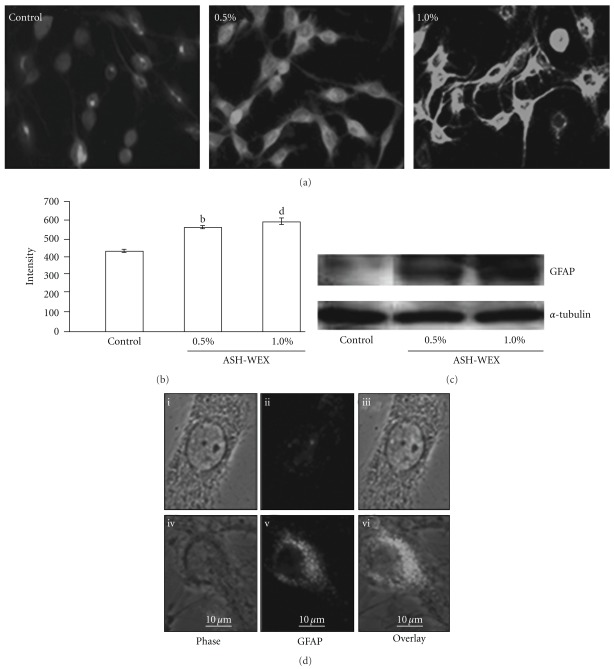
GFAP expression in response to ASH-WEX treatment. (a) Immunofluorescence detection of glial differentiation marker, GFAP, is shown in control, ASH-WEX (0.5 and 1.0%)-treated C6 glioma cultures. Images were captured using Nikon E600 fluorescent microscope. The relative intensity measurement of immunofluorescence is shown (b). Compared with control, significant changes were found in ASH-WEX-treated cells as seen by (b) (*P* < .02) and (d) (*P* < .001). (c) Representative western blot hybridization signals for GFAP from control and test samples (0.5 and 1.0% ASH-WEX-treated). (d) Single cell micrographs of GFAP immunofluorescence in control (i–iii) and 0.5% ASH-WEX-treated cultures (iv–vi).

Human YKG1, A172, U118MG and rat C6 cell lines when treated with 0.5% of ASH-WEX, showed morphological changes as shown in Figure 1. They showed differentiated morphology with increased number of processes and enlarged cell size. The cells were growth inhibited and exhibited stellate process formation indicative of a more differentiated astrocytic phenotype. In order to confirm the occurrence of differentiation, we examined the expression of differentiation marker, GFAP, and found that the ASH-WEX treatment leads to increased expression of GFAP (Figure 5). The ASH-WEX-treated cells showed enhanced GFAP immunoreactivity and were having multiple processes and enlarged cell size as compared to the control cells (Figures 5(a) and 5(d)). Treatment of cells with ASH-WEX doses >1.0% caused vacuolization in the cytoplasm, retraction, rounding and detachment of cells from the surface (Figure 1). Increase in GFAP expression in 0.5 and 1.0% treated cells was also supported by western blotting results.

**Figure 6:**
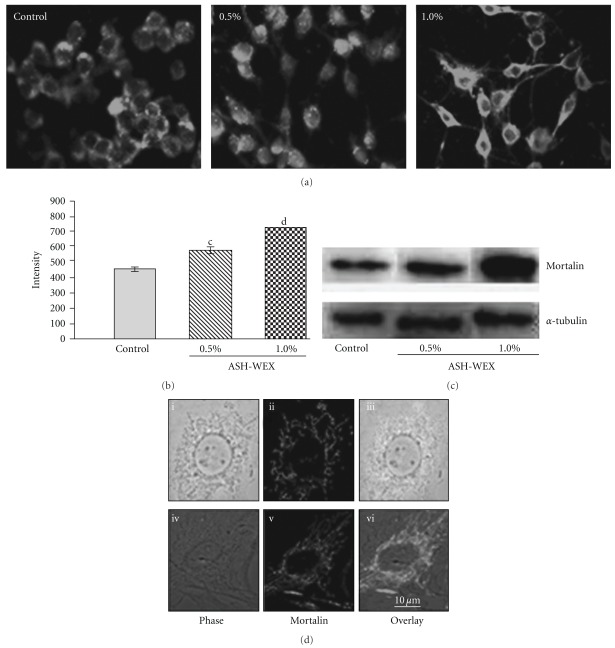
Mortalin in response to ASH-WEX treatment. (a) Immunofluorescence detection of mortalin is shown in control, 0.5 and 1.0% ASH-WEX-treated C6 glioma cultures. Images were captured using Nikon E600 fluorescent microscope. (b) Relative intensity measurement of immunofluorescence. Significant change in mortalin expression in response to ASH-WEX-treated cultures compared with control is seen by (c) (*P* < .01) and (d) (*P* < .001). (c) Representative western blot hybridization signals for mortalin from control and test samples (0.5 and 1.0% ASH-WEX-treated cells). Single cell micrographs of mortalin immunofluorescence in control (i–iii) and 0.5% ASH-WEX-treated (iv–vi) cultures.

Based on the growth arrest induced by ASH-WEX in glioma cells, we next examined whether its effect caused induction of senescence in C6 glioma cells. The control and ASH-WEX-treated cells were stained for mortalin (Figure 6) that has been previously shown to change the subcellular localization upon induction of senescence [13]. We found that the ASH-WEX-treated cells that attained differentiated morphology also showed pancytoplasmic staining typical of the normal cells. The shift in mortalin staining pattern occurred in >80% cells treated with low concentration of ASH-WEX (0.1–0.5%) (Figures 6(a) and 6(d)). The staining intensity of mortalin was found to be significantly increased in ASH-WEX-treated cells, further confirmed by western blotting results (Figure 6(c)). 

### 3.5. Change in Cell Adhesion Properties

**Figure 7:**
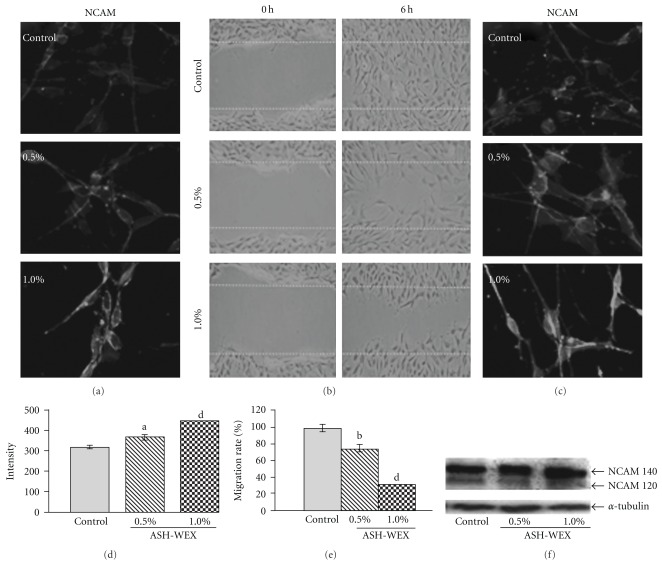
NCAM detection in the presence of ASH-WEX. Immunofluorescence detection of NCAM is shown in control, 0.5 and 1.0% ASH-WEX-treated C6 glioma cultures (a). Images were captured using Nikon E600 fluorescent microscope. Representative phase contrast images of control, 0.5 or 1.0% ASH-WEX-treated cells, in which motility was analyzed by wound-scratch test. Images show the starting (0 hours after scratch) and the end (6 hours after scratch) point of the analysis (b). Immunocytochemistry of NCAM analysis performed in both control and in ASH-WEX-treated cells at 6 hours post wounding (c). Graph showing the relative intensity measurement of immunofluorescence after 72 hours of treatment with ASH-WEX (d). (e) Graph shows that the rate of C6 glioma migration in response to ASH-WEX treatment in comparison to untreated cells. Data are obtained from a set of scratch test analysis (*N* = 3) and are expressed as means ± standard error. Representative western blot hybridization signals for NCAM from control and test samples (0.5 and 1.0% ASH-WEX treated for 72 hours) (f). a: (*P* < .05), b: (*P* < .02) and d: (*P* < .001) are significant changes in ASH-WEX-treated cultures as compared with control cultures.

Since brain tumors have the characteristics of being highly invasive, we next examined the invasion property of glioma cells as consequent to the treatment with ASH-WEX. The expression level of cell adhesion marker NCAM-120 and -140 isoforms significantly increased in cells treated with ASH-WEX, as detected by both immunostaining and western blot analyses (Figures 7(a), 7(d) and 7(f)). To evaluate the motility of C6 glioma cells, we analyzed them in wound healing assay with/without ASH-WEX. As shown in Figure 7(b), untreated C6 glioma cells were able to invade the scratched area that was fully re-colonized by 6 hours. The treatment with 0.5 and 1.0% water extract strongly reduced the migration rate of the C6 glioma cells. In fact, 6 hours after the scratch, very few cells were seen in the scratched area in the 1.0% treatment group (Figure 7(b)). Quantitative analysis also indicated a significant decrease (*∼*35–75%) in cell migration rate following Ashwagandha treatment (Figure 7(e)). Furthermore, immunostaining study revealed that all the cells migrating to the scratched area showed enhanced NCAM expression in ASH-WEX-treated group as compared with the control group (Figure 7(c)). 

## 4. Discussion

**Figure 8:**
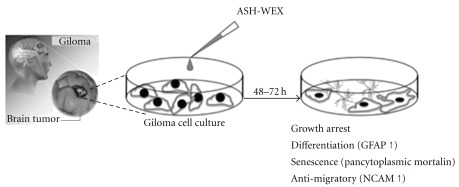
Hypothetical diagram summarizing the anti-proliferative, differentiation- and senescence-inducing, anti-migratory effects of the ASH-WEX.

Glioma is one of the most common primary brain tumors with only limited options for treatment, and hence calls for the development of novel therapeutic approaches. Alcoholic extract of Ashwagandha leaves was shown to be selectively cytotoxic to multiple human cancer cells [4]. Recently, immune modulatory and apoptosis-inducing properties of the leaf extract of Ashwagandha have been reported [15, 16]. In the present study, we provide evidence that the ASH-WEX also has anti-proliferative, differentiation-inducing and anti-migratory activity for human and rat glioma cells. The results are summarized in Figure 8 hypothetical diagram. ASH-WEX has several advantages relating to its feasibility and ease of preparation, use and consumption as an Ayurvedic supplement or medicine. We found that the treatment of C6 glioma, YKG1, U118MG and A172 cells with ASH-WEX (0.5 and 1.0%) for 72 hours significantly inhibited their proliferation. There was a concentration-dependent decrease in the cell viability as assessed by trypan blue dye exclusion analysis and MTT assay with ASH-WEX, concentration >1.0% being cytotoxic. Thus, ASH-WEX displayed strong anti-proliferative activity in C6 glioma cells with an IC50 of about 1.25%. Growth inhibition of many human tumor cell lines, such as human osteogenic sarcoma, fibrosarcoma, breast carcinoma, lung carcinoma and colon carcinoma and normal cells has been reported with Ashwagandha extracts [1]. The anti-proliferative activity of ASH-WEX in human and rat glioma cells may be the result of inhibition of proliferation and differentiation of tumor cells as verified by MTT test (Figure 2) and enhanced expression of GFAP (Figure 5), a marker of glial cell differentiation. These results are further supported by phase contrast images of glioma cells (Figure 1) that show the correlation between the treatment with ASH-WEX (0.5 and 1.0%) and reduction in the number of cells as well as their differentiated morphology. 

To evaluate the possible nature of bioactive molecules in the ASH-WEX, the control and heat, proteinase K, trypsin, DNase and RNase-treated ASH-WEX were tested for anti-proliferative activity using MTT assay. There was no significant difference in the anti-proliferative activity of the extract after different treatments, suggesting that the active components are neither heat labile, nor proteinecious and nor nucleic acid in nature. The possibility of the active molecule being a lipid was also excluded, as ASH-WEX is aqueous in nature. Earlier studies with the root extract of Ashwagandha have characterized water-soluble fractions to contain sitoindosides VII-VIII and withaferin A [17]. Interestingly, withaferin A has been found to be present in the alcoholic extracts of Ashwagandha leaves [4]. Withaferin A has been well studied for its tumor-inhibitory potential both *in vitro* and *in vivo* studies [4, 18–20]. Its presence along with other possible bioactive components in aqueous leaf extract is yet to be established by further studies.

The astrocyte marker, GFAP, is involved as a collaborator in the complex cellular processes controlling astrocytoma cell morphology, differentiation and proliferation [21]. GFAP was found to be increased in C6 glioma cells following ASH-WEX treatment, as observed by immunostaining and western blotting results. C6 cells transfected with GFAP cDNA showed significantly reduced tumor growth [21]. The growth and invasive potential of the antisense GFAP-transfected astrocytoma cells was significantly enhanced [22]. Some other studies have also detected marked increase in GFAP and induction of differentiation with various differentiating agents such as neomycin [23], dimethylformamide [24], tanshione [25] and retinoic acid [26]. Our data suggested that the ASH-WEX is able to induce differentiation in C6 glioma cells, indicating it to be a potential differentiation-inducing and anti-cancer agent in glioma. Several recent studies have reported that the induction of cellular differentiation is an attractive therapeutic strategy against glioma cell tumorigenicity [27]. These investigations support our current results showing the limited proliferation and reversion of phenotype associated with enhanced GFAP expression in ASH-WEX-treated cultures. Induction of differentiation by ASH-WEX may be further explored as a novel approach in the multimodality treatment of gliomas.

The differentiation-inducing potential of ASH-WEX was further confirmed by immunostaining pattern of mortalin protein in control and treated cells. Mortalin is distributed in a pancytoplasmatic manner in normal cells, but in immortal cells its localization shifts to the perinuclear zone [13, 28]. In the transformed cells, the reversion of subcellular distribution of mortalin from perinuclear to pancytoplasmic type has been reported to correlate with induction of senescence by introduction of a single chromosome, chromosome-fragments, and genes or chemicals [29, 30]. Consistent with these observations, whereas the control C6 glioma cells showed perinuclear localization of the mortalin protein, the ASH-WEX-treated cultures showed pancytoplasmic localization. The data further indicated that the glioma cells that undergo differentiation by ASH-WEX treatment become non-proliferative. We also found that there was a significantly higher level of mortalin expression in the 1.0% treated group as compared to the control and 0.5% ASH-WEX treatment group. Such increase in mortalin may represent a stress response to the ASH-WEX treatment, an adaptive response. Mechanism and significance of such increase in mortalin in ASH-WEX-treated differentiated cells remains to be elucidated. In an earlier study, mortalin expression was detected in neurons in contrast to the glial and astrocytes in normal brain tissues [31]. The level of mortalin expression has been shown to decrease in Parkinson's disease (PD) patients signifying that it has a role in PD [32, 33]. In light of these reports and our data that mortalin is induced in ASH-WEX-treated glioma cells that showed differentiated phenotype, we predict that mortalin has some novel functions in an induction or maintenance of neuronal and glial differentiation. Molecular mechanism(s) of these functions, their role and significance in disease therapy warrant further studies.

The present data also indicated that the C6 glioma cells that express NCAM on their cell surface, show reduced proliferation in response to ASH-WEX treatment. NCAM has been implicated in cell-cell interaction throughout the nervous system [34] and is expressed by many tumors of neuroectodermal origin, including astrocytic tumors [35, 36]. In addition to cell-cell adhesion, homophilic interaction of NCAM induces signal transduction, resulting in neuronal differentiation [37] and inhibition of cell proliferation [38, 39]. Moreover, expression of NCAM has been shown to reduce migration and invasion of glioma cells *in vitro* as well as *in vivo* [40–42]. Western blot of NCAM in ASH-WEX-treated C6 glioma cells showed significant increase in the expression of NCAM 140 isoform. Expression of the transmembrane 140-kDa isoform of NCAM has been shown to cause significant reduction in cellular motility [43]. The data were further supported in the present anti-motility assay study in which 1.0% WEX-treated cells were least motile, whereas control cells migrated to the scratched area within 6 hours of treatment and covered the scratched area. Migration can be correlated with *in vivo* invasiveness studies. Published evidence indicates that the reduction in NCAM expression is correlated with increased tumor invasiveness and overexpressing the transmembrane isoform NCAM 140 in an invasive NCAM-negative variant of the glioma inhibited cell invasion [44, 45].

Thus the enhanced expression of GFAP, mortalin and NCAM in ASH-WEX-treated cells, in the present study, may suggest the possible mechanism(s) of differentiation inducing potential of Ashwagandha for the treatment of gliomas. Similar effects have been observed with alcoholic extract and its purified components (Withaferin A, Withanone, Withanolide A) in C6 glioma cells [20]. Since polysialylated (PSA) form of NCAM has been implicated to facilitate tumor invasiveness [46], further studies on its expression pattern in response to Ashwagandha treatment will be of great interest.

Although Ashwagandha is most commonly used in Indian Ayurvedic medicine, the mechanistic aspects of its effects are still unknown and potential of its bioactive components are yet to be recognized. Most of the reported differentiating agents in glioma (retinoids) are heat-labile and water insoluble. Thus evaluation and characterization of the water-soluble active components for discovery of potentially safe glioma-therapeutic phyto-reagents is warranted.

## Funding

The study was partly supported by grants from the Department of Biotechnology, Government of India to Guru Nanak Dev University, India; and the International Affairs Department, AIST, Japan under AIST (Japan)-DBT (India) bilateral collaboration and from the NEDO (New Energy and Industrial Technology Development Organization) of Japan. H. K. is supported by fellowship grant from the Council of Scientific and Industrial Research (CSIR), India. N. S. is a recipient of AIST International Fellowship and MEXT Scholarship, Japan.

## Figures and Tables

**Table 1:** Analysis of phytochemicals in the ASH-WEX.

Phytochemicals	ASH-WEX
Flavonoids	+
Steroids	+
Tannins	+
Anthroquinones	−
Triterpenoids	−
Amino acids	+
Saponins	+
Phytosterols	−
Reducing sugars	+
Alkaloids	
Hager's test	+
Marqui's test	+

“+": presence; “−": absence.
